# Ultrastructural Characteristics of Neuronal Death and White Matter Injury in Mouse Brain Tissues After Intracerebral Hemorrhage: Coexistence of Ferroptosis, Autophagy, and Necrosis

**DOI:** 10.3389/fneur.2018.00581

**Published:** 2018-07-17

**Authors:** Qian Li, Abigail Weiland, Xuemei Chen, Xi Lan, Xiaoning Han, Frederick Durham, Xi Liu, Jieru Wan, Wendy C. Ziai, Daniel F. Hanley, Jian Wang

**Affiliations:** ^1^Department of Anesthesiology and Critical Care Medicine, Johns Hopkins University School of Medicine, Baltimore, MD, United States; ^2^Department of Biochemistry and Molecular Biology, School of Basic Medical Sciences, Capital Medical University, Beijing, China; ^3^Advanced Innovation Center for Human Brain Protection, Beijing, China; ^4^Department of Human Anatomy, College of Basic Medical Sciences, Zhengzhou University, Zhengzhou, China; ^5^Department of Neurology, Johns Hopkins University School of Medicine, Baltimore, MD, United States

**Keywords:** cell death, intracerebral hemorrhage, synapse, transmission electron microscopy, white matter injury

## Abstract

Although intracerebral hemorrhage (ICH) is a devastating disease worldwide, the pathologic changes in ultrastructure during the acute and chronic phases of ICH are poorly described. In this study, transmission electron microscopy was used to examine the ultrastructure of ICH-induced pathology. ICH was induced in mice by an intrastriatal injection of collagenase. Pathologic changes were observed in the acute (3 days), subacute (6 days), and chronic (28 days) phases. Compared with sham animals, we observed various types of cell death in the injured striatum during the acute phase of ICH, including necrosis, ferroptosis, and autophagy. Different degrees of axon degeneration in the striatum were seen in the acute phase, and axonal demyelination was observed in the ipsilateral striatum and corpus callosum at late time points. In addition, phagocytes, resident microglia, and infiltrating monocyte-macrophages were present around red blood cells and degenerating neurons and were observed to engulf red blood cells and other debris. Many synapses appeared abnormal or were lost. This systematic analysis of the pathologic changes in ultrastructure after ICH in mice provides information that will be valuable for future ICH pathology studies.

## Introduction

Intracerebral hemorrhage (ICH) accounts for 15% of the 15 million annual stroke cases worldwide (1). Only 60% of patients with ICH survive 1 month past initial onset (2, 3). Most survivors are left with disability, as only 12–39% regain functional independence (3). However, research into ICH is outpaced by research into ischemic stroke (4).

ICH develops when ruptured blood vessels cause extravasation of blood into surrounding brain tissue. Bleeding is followed by erythrophagocytosis, or red blood cell lysis, and the release of hemoglobin (5), which then leads to iron toxicity (6–8), cell death, and inflammation (9–11). Studies have shown that various types of cell death can occur after ICH, including apoptosis (12–14) and necrosis (15–17) in humans and rodents, and autophagic cell death (18, 19) and ferroptosis in animal models (5, 20). In addition, an elevated inflammatory response is associated with recruitment of resident microglia and infiltrating monocyte-derived macrophages (1, 21). However, the ultrastructural pathology of the inflammation has not been clearly characterized in animal models (22). Besides neuronal death and the inflammatory response in the acute phase of ICH, axonal damage, including demyelination (16, 23, 24), and synapse changes (25) also contribute to ICH pathology and may play vital roles in long-term outcomes such as mood and cognition changes (26–28).

Electron microscopy provides a valuable means of analyzing and characterizing cerebral sections of subjects after ICH (16). In human ICH studies, transmission electron microscopy (TEM) reveals cell swelling (including neurons, endothelia, and astrocytes), focal cell necrosis, organelle disruption, and mitochondrial breakdown (16). In animal models of subarachnoid hemorrhage, a widening of inter-endothelial tight junctions has been reported (29). Subarachnoid hemorrhage also has been associated with structural changes in endothelial cell vacuoles, erythrocytes, leukocytes, and mast cells, and with cellular necrosis (30, 31). In animal models of ICH, neuronal damage, nucleolus condensation, mitochondrial swelling, and synapse destruction have been reported (5, 12, 13, 18, 25). However, no study has systematically examined the ultrastructural features of collagenase-induced ICH in mice over time. The goal of this study was to investigate pathologic changes in the ultrastructure of neural soma, axons, synapses, myelination, and innate immune cells using TEM during the acute and chronic phases of ICH. We hypothesized that neuronal death, axonal degeneration, and synaptic degeneration would be observed in the perihematoma region in the subacute phase, and that demyelination of axons would be present in the chronic phase after ICH. The insight gained from this study will advance our knowledge of ICH-induced ultrastructural changes and will be essential for planning future ICH studies.

## Materials and methods

### Animals

Twelve-week-old C57BL/6 male mice were obtained from Jackson Laboratories. A total of 24 animals were used in this study. Animals and slice cultures for each group were randomized with the website www.randomization.com (32, 33). This study was carried out in accordance with the recommendations of ARRIVE guidelines. The protocol was approved by the Johns Hopkins University Animal Care and Use Committee.

### ICH model

Mice were anesthetized with 1–3% inhaled isoflurane and ventilated with oxygen-enriched air (20%:80%). They were injected in the left striatum with 0.5 μL of 0.075 U collagenase VII-S (Sigma-Aldrich) at 0.1 μL/min followed by a 5 min pause. Injections were administered at 0.8 mm anterior and 2.1 mm lateral of the bregma, and 3.1 mm in depth on a stereotactic instrument with an automated injector (5). Sham-operated mice received needle insertion only.

### TEM

Sham and ICH mice at 3, 6, and 28 days of recovery were perfused with 2% paraformaldehyde and 2% glutaraldehyde in 0.1 M sodium cacodylate buffer, followed by post-fixation in 2% osmium tetroxide with 1.6% potassium ferrocyanide in 0.1 mol/L sodium cacodylate (5). We cut 5-mm^3^ samples from the margin of the hematoma (days 3 and 6 samples), the margin of the glial scar (day 28 samples or corresponding location in sham mice), and the corpus callosum (near the glial scar or corresponding location in sham mice), stained them *en bloc* with 2% uranyl acetate, dehydrated them in ethanol, and embedded them in eponate. We stained semi-thin sections with hematoxylin and eosin to identify the orientation and location (margins of hematoma or glial scar) of the sections under a microscope. Then the sections (70–90 nm) were placed on copper slot grids and stained with 2% uranyl acetate and lead citrate. Images were captured with a Hitachi H7600 TEM in the microscope core of Johns Hopkins University and Capital Medical University.

In this study, 693 images were taken for the cell death study, and mitochondrial area was examined in approximately 172 neuronal soma and 956 axons; 231 images were taken for reviewing synaptic changes; and 151 images were taken for quantifying axonal changes in sham and ICH mice (subacute phase). Approximately 2987 axons in 467 images were examined to enable quantification of demyelination in both striatum and corpus callosum in sham and ICH mice (chronic phase). Data were analyzed in blinded fashion on coded brain sections.

We measured the area of all mitochondria in neuronal soma and axons in all of the images that we took. The data are presented as frequency of distribution of the area of mitochondria. We determined the synapse density by counting the total number of synapses in one area at 24,500 × magnification (100 μm^2^). Active area of synapse was measured as the area of active zone in each synapse. Docked vesicles in presynapses were calculated as the number of visible vesicles in each presynapse. Axon diameter was determined as the outer diameter of each axon in all images. Axon density was calculated by determining the number of axons in an area at 24,500 × magnification (1 μm^2^). The percentage of unmyelinated axons was quantified by determining the number of unmyelinated axons as a percentage of the total number of axons.

### Fuoro-Jade C staining

Fluoro-Jade C (FJC) was used to identify degenerating neurons in the acute stage of ICH as previously described (5). Brain sections were observed and photographed under a fluorescence microscope at an excitation wavelength of 450–490 nm.

### Statistical analysis

Data are presented as mean ± SD, bar graph, or dot plot. We made two-group comparisons with a two-tailed Student's *t*-test followed by Welch's correction. One-way ANOVA followed by Dunn's *post-hoc* analysis was used to determine where those differences occurred among groups. All analyses were carried out with GraphPad Prism 5.0 (GraphPad Software, Inc.,). The criterion for statistical significance was *p* < 0.05.

## Results

### Neurons and microglia in the brains of sham animals

**Figure 1:**
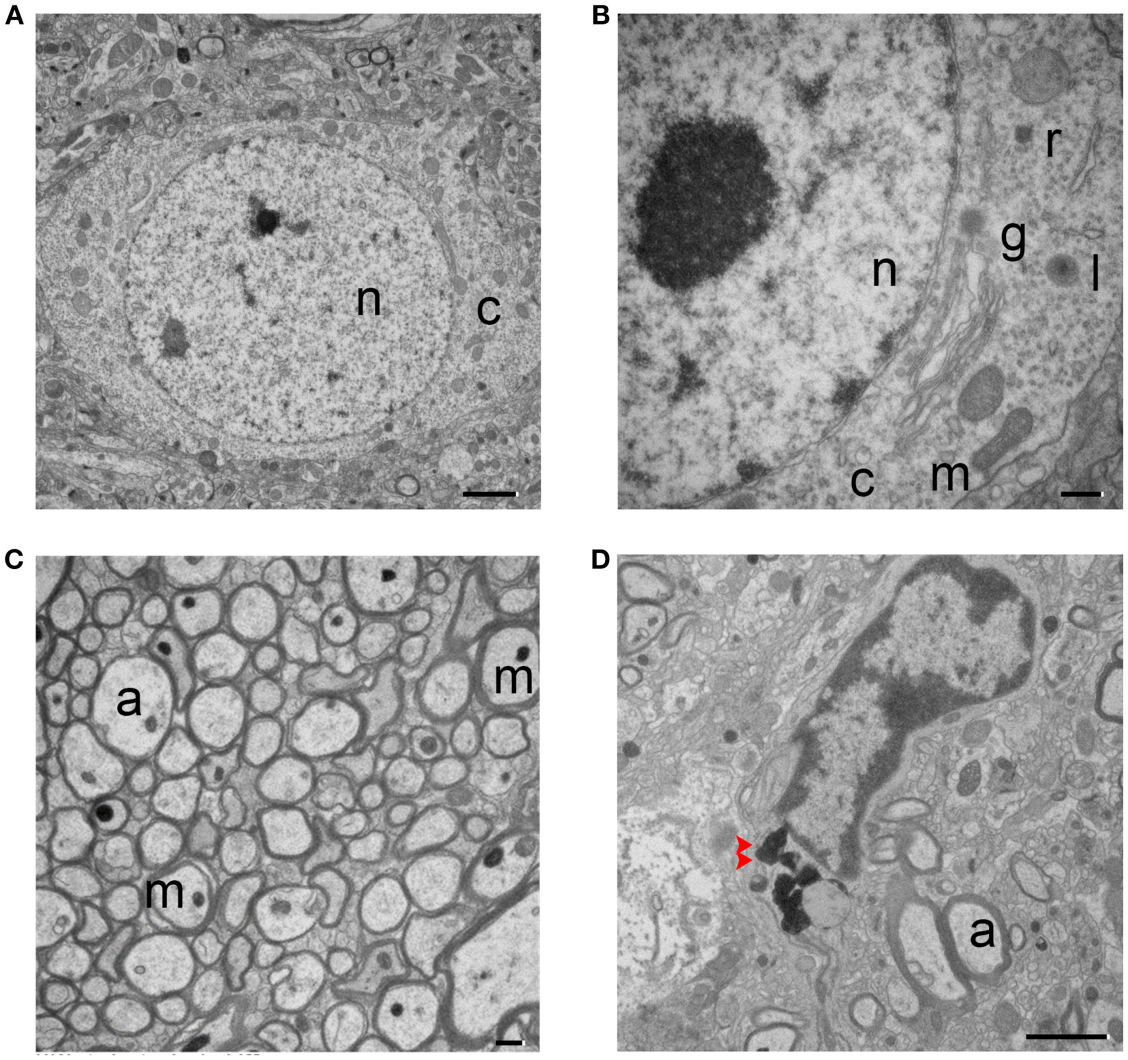
Transmission electron micrographs of striatum from sham mouse brain tissue. **(A,B)** The structure of the neuronal soma is visible with normal mitochondria (m), nucleus (n), and cytoplasm (c). The higher magnification image in **(B)** shows additional organelles, including Golgi body (g), lysosome (l), and ribosome (r). **(C)** Healthy, myelinated axons. Axons (a) are tightly wrapped by myelin, and axonal mitochondria (m) and neurofilaments are present. **(D)** A microglial cell is shown. Dark inclusions are indicated with red arrow heads. Scale bars: **(A,D)** 2 μm; **(B,C)** 500 nm. *n* = 6 animals per group.

Striatal tissue from sham animals showed intact and healthy neuronal soma (Figures 1A,B). The nuclear envelope was intact, several distinct nucleoli were present, and DNA showed normal compaction. Organelles, including the Golgi body, lysosomes, ribosomes, and mitochondria were visible and exhibited normal characteristics. Neuronal axons were relatively uniform in size and shape with an intact circle patch. Myelin tightly wrapped most axons, mitochondria had a healthy morphology, and dot-shaped neurofilaments were visible in most axons (Figure 1C). Microglia were also present (Figure 1D); they exhibited distinct heterochromatin close to the nuclear membrane and a small volume of plasma, indicating a relatively quiescent condition. Dark inclusions in the cytoplasm might be engulfed myelin debris.

### Forms of neuronal death in the ICH brain

**Figure 2:**
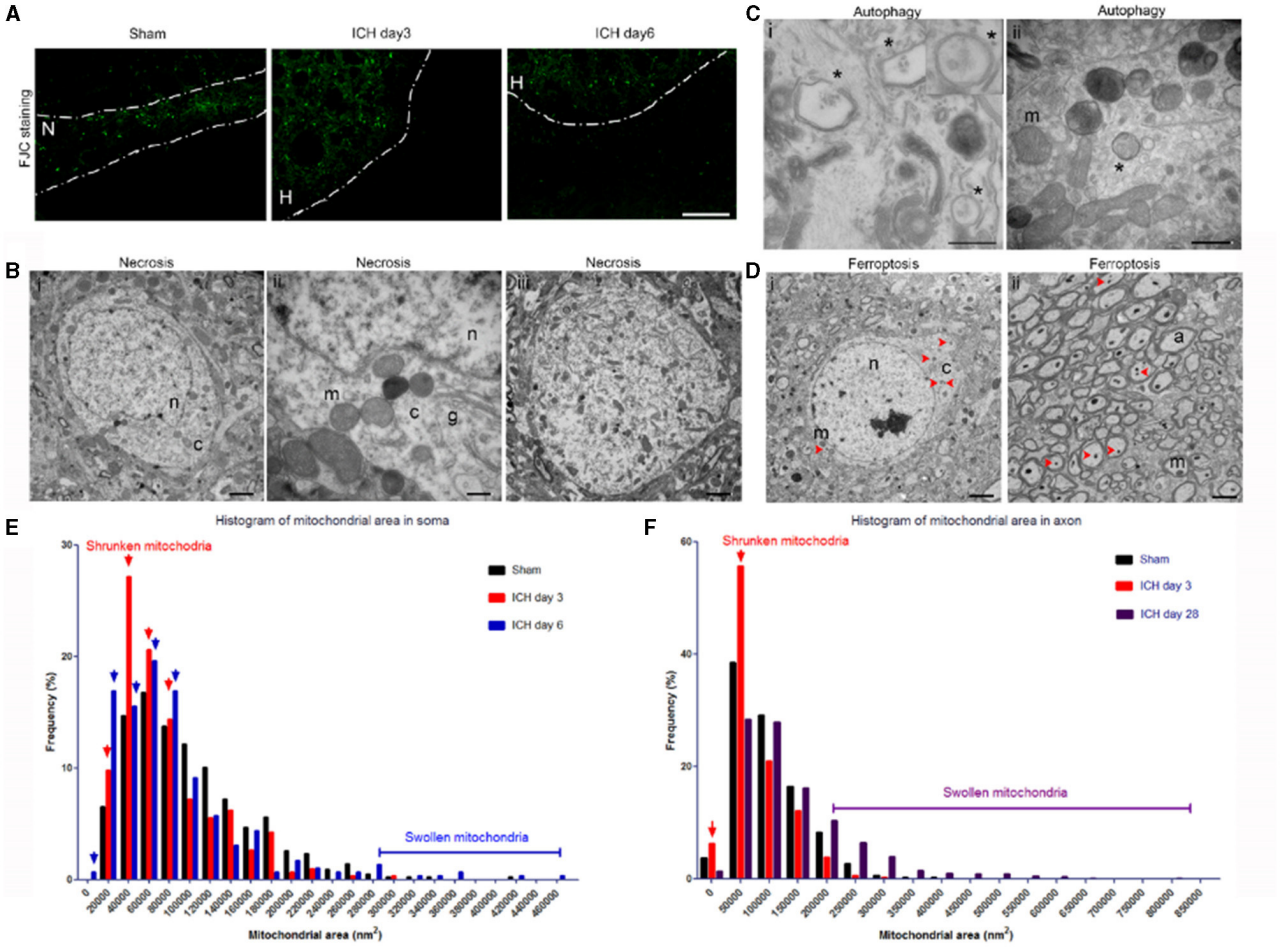
Mixed forms of neuronal death after intracerebral hemorrhage (ICH). **(A)** Fluoro-Jade C histology shows degenerating neurons in the perihematoma region at 3 and 6 days post-ICH or along the needle track in the striatum of the sham-operated mice. Dashed lines indicate the margin of hematoma. N, needle track; H, hematoma. **(B)** Necrotic neuronal soma (i–iii) show loss of a distinct nuclear membrane, enlarged mitochondria, and changes in chromatin structure at 3 days post-ICH. **(C)** Autophagy in neuronal soma at 3 days post-ICH; autophagosomes are labeled with asterisks. Inset shows a high-power image of an autophagosome. **(D)** Neuronal soma and axons show signs of ferroptosis with evidence of shrunken mitochondria (red arrowheads) at 3 days post-ICH. **(E)** Quantification of mitochondrial area frequency in neuronal somas at various time points after ICH. Arrows indicate increased frequency of shrunken mitochondria on days 3 and 6. Number of mitochondria: sham, *n* = 429; ICH day 3, *n* = 306; ICH day 6, *n* = 296. **(F)** Quantification of mitochondrial area frequency in axons at various time points after ICH. Arrows indicate increased frequency of shrunken mitochondria on day 3. Number of mitochondria: sham, *n* = 433; ICH day 3, *n* = 314; ICH day 28, *n* = 603. Scale bars: **(A)** 100 μm; **(Bi,iii, D)** 2 μm; **(Bii, Cii)** 500 nm. n, nucleus; c, cytoplasm; m, mitochondria; a, axon. *n* = 6 animals per group.

Using standard histology and immunostaining methods such as FJC-, TUNEL-, and propidium iodide-staining, neuronal death can be detected as early as 2 h after thrombin-induced ICH (34) or 6 h after collagenase-induced ICH (5), and is present up to 7 days (5). In our collagenase-induced ICH model, we observed FJC^+^ cells in the perihematoma region at days 3 (acute phase) and 6 (sub-acute phase) (Figure 2A). Consistent with this finding, several forms of neuronal death were observed with TEM in the same brain region at 3 and 6 days post-ICH. The neuronal somas exhibited loss of a distinct (Figures 2Bi,ii) or total loss (Figure 2Biii) of the nuclear envelope, and mitochondria and other organelles were enlarged and swollen, indicating different degrees of necrosis. Autophagic neurons were seen with autophagosomes, which appeared as double-membrane–bound autophagic vesicles in somas (Figure 2C). However, we failed to observe morphologic signs of classic apoptosis as identified by heterochromatin (chromatin condensation) and apoptotic bodies (35).

Importantly, the newly identified cell death form ferroptosis was also seen in the same animals in both somas and axons (Figures 2D–F). The mitochondria of ferroptotic cells appeared shrunken when compared with those in sham tissue, as evidenced by increased frequency of smaller mitochondrial area (Figures 2D–F). We quantified the mitochondrial area in neuronal soma (no morphologic evidence of classic necrosis, apoptosis, and autophagy) of animals 3 and 6 days post-ICH, as well as that of sham animals to confirm the presence of ferroptotic cells. We found increased frequency of small mitochondrial area at both 3 days (Figure 2E, red arrows) and 6 days (Figure 2E, blue arrows) compared to that in sham animals; at 6 days, some mitochondria were larger than those of sham mice. We also investigated whether ferroptotic neurons were still present on day 28 post-ICH. Because FJC staining failed to detect degenerating neuronal soma later than 7 days, we quantified and analyzed only the mitochondrial area in axons. As expected, and consistent with our previous findings (5), the frequency of shrunken mitochondria was increased at day 3 (Figure 2F, red arrows), but not at day 28, compared to that in sham animals. Interestingly, swollen mitochondria were still largely present at day 28 (Figure 2F). Unfortunately, without specific markers to detect ferroptosis, we were unable to compare results from TEM to those from immunostaining.

### Axonal degeneration in the ICH brain

**Figure 3:**
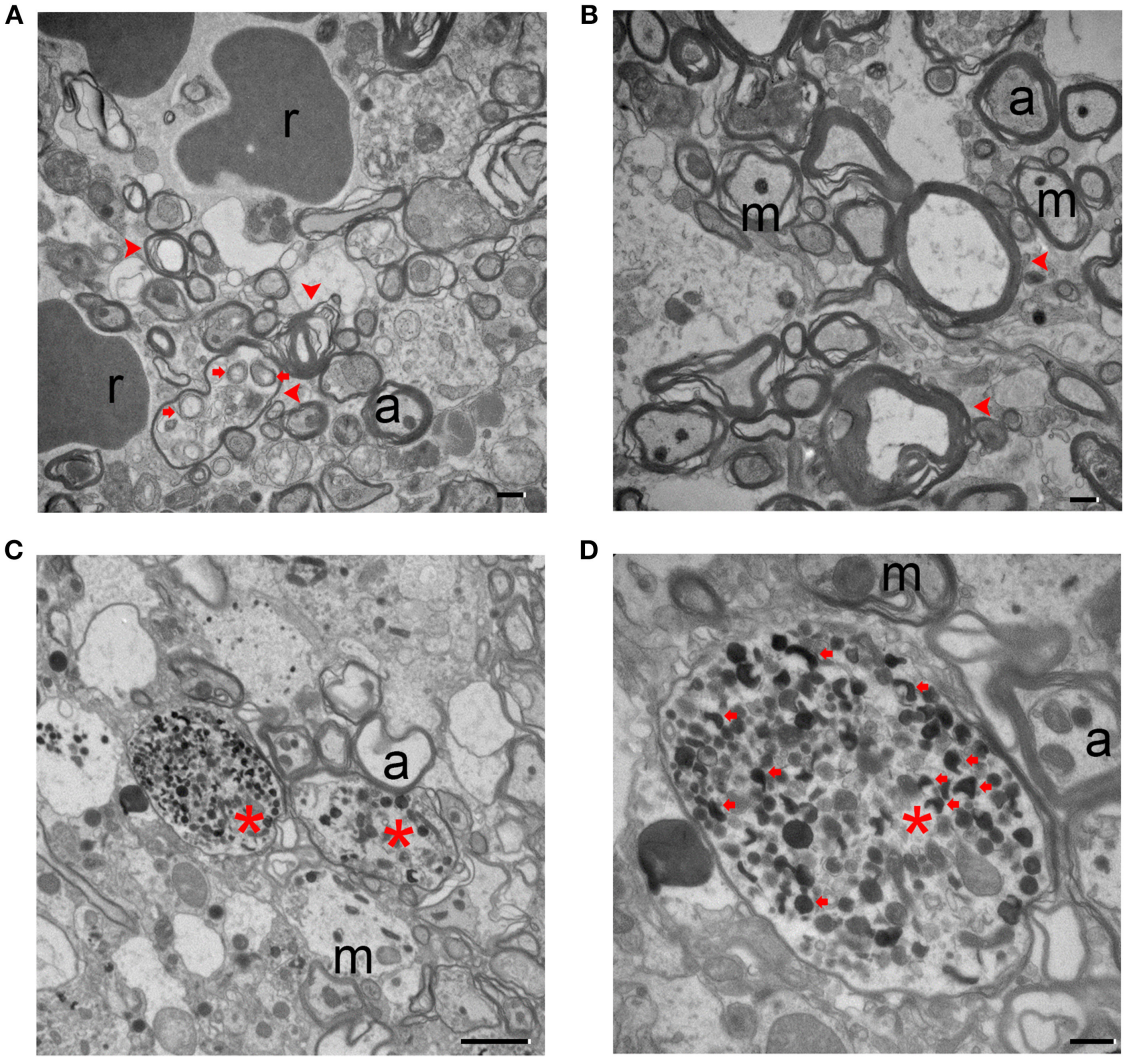
Degenerating axons at 3 days after intracerebral hemorrhage. **(A,B)** The axons show a severe loss of uniformity. Degenerating axons are indicated with red arrowheads. There is clear disruption of the myelin sheath and organelles, including autophagosomes (red arrows). **(C,D)** Dystrophic neurites (red arrows) are present in degenerating axons (red asterisks). A higher power image is shown in **(D)**. Scale bars: **(A,B,D)** 500 nm; **(C)** 2 μm. r, red blood cell; m, mitochondria; a, axon. *n* = 6 animals per group.

Neurofilament damage can be detected by immunostaining as early as 1 day post-ICH. Additionally, axonal bundles are fragmented and barely detectable at 7 days, but regenerate by 14 days (24). In our study, injured animals displayed different forms and degrees of axonal degeneration under TEM as early as day 3 post-ICH. As shown in Figure 3, axons no longer exhibited a uniform size and shape, and tissue from injured mice had fewer axons than tissue from sham mice (Figure 1C). Myelin on most of the axons appeared fractured (Figures 3A,B). As opposed to the healthy mitochondria and neurofilaments seen in the uninjured brain (Figure 1C), ICH brain tissue exhibited swollen cytoplasm and degenerative neurofilaments. Mitochondria and other organelles appeared swollen or dark and dense as has been reported in ischemic stroke animal models (36). Axons contained autophagosomes (Figures 3A–D), and neurites were abnormally enlarged or swollen (Figures 3C,D). These classic signs of dystrophy indicate the presence of severe cell death and/or axonal degeneration.

### Microglia and infiltrating macrophages in the perihematoma region of the ICH brain

We and others have shown that activated Iba-1^+^ microglia/macrophages are in the perihematoma region as early as 1 h after ICH (37–39). However, because the expression markers for microglia and infiltrating macrophages are similar, these two cell populations are difficult to identify and differentiate by immunostaining; moreover, it is not easy to observe engulfment of red blood cells and degenerating neurons by microglia/macrophages *in vivo*.

**Figure 4:**
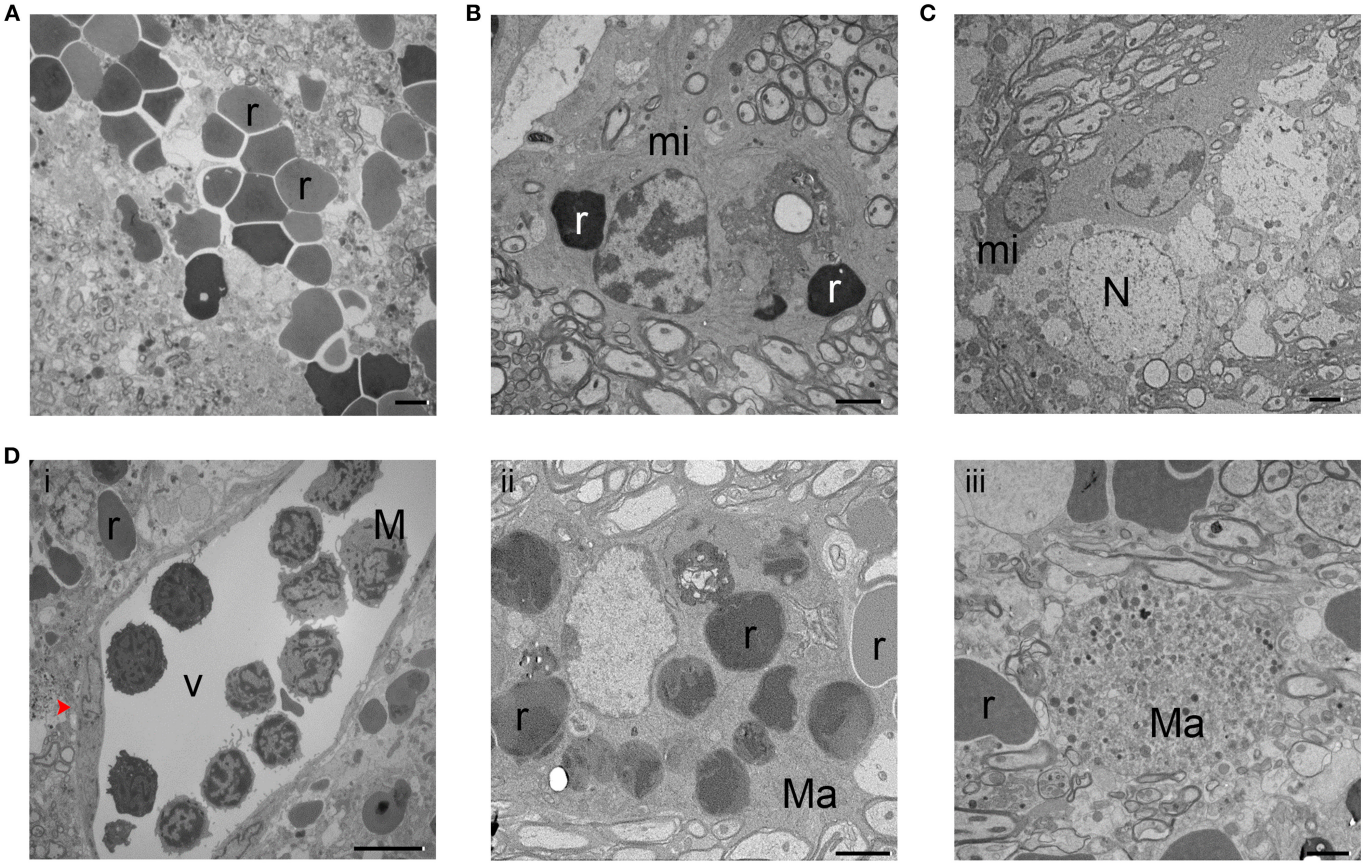
Microglia and monocyte-derived macrophages at 3 days after intracerebral hemorrhage. **(A)** Bleeding in the brain tissue is evident by the presence of red blood cells (r). **(B,C)** Reactive phagocytic microglia (mi) are shown engulfing red blood cells (r) and necrotic neurons (N). **(D)** A blood vessel (V) is shown with white blood cells, including monocytes (M). Red arrowhead indicates a blood vessel endothelial cell (i). Macrophages (Ma) can be seen phagocytosing red blood cells (r) (ii) and other debris (iii). Scale bars: **(A–C,Dii,iii)** 2 μm; **(D)** 10 μm. *n* = 6 animals per group.

TEM revealed extravasated red blood cells at 3 days post-ICH (Figure 4A). Resident microglia were recruited to the peri-hematoma region and exhibited reactive morphology (Figures 4B,C), including enlarged nuclei and cytoplasm and longer branches between cells and axons. Notably, we captured a microglial cell that was engulfing red blood cells and other debris (Figure 4B) and two microglia that were present near the site of necrotic neurons (Figure 4C).

Monocytes and other white blood cells were seen in a blood vessel (Figure 4Di) (40). Monocyte-derived macrophages were seen in brain parenchyma engaging in phagocytosis of red blood cells (Figure 4Dii) and other cell debris (Figure 4Diii), which appear as small dark bodies in the cytoplasm.

### Synaptic changes in the ICH brain

Few studies have examined synaptic changes in the ICH brain. In one prior study, Nguyen et al. observed dendritic atrophy in Golgi-Cox–stained neurons in the perihematoma region at day 7, but not at day 60; interestingly, they also observed dendritic arborization in the contralateral striatum at days 7 and 60 (41).

**Figure 5:**
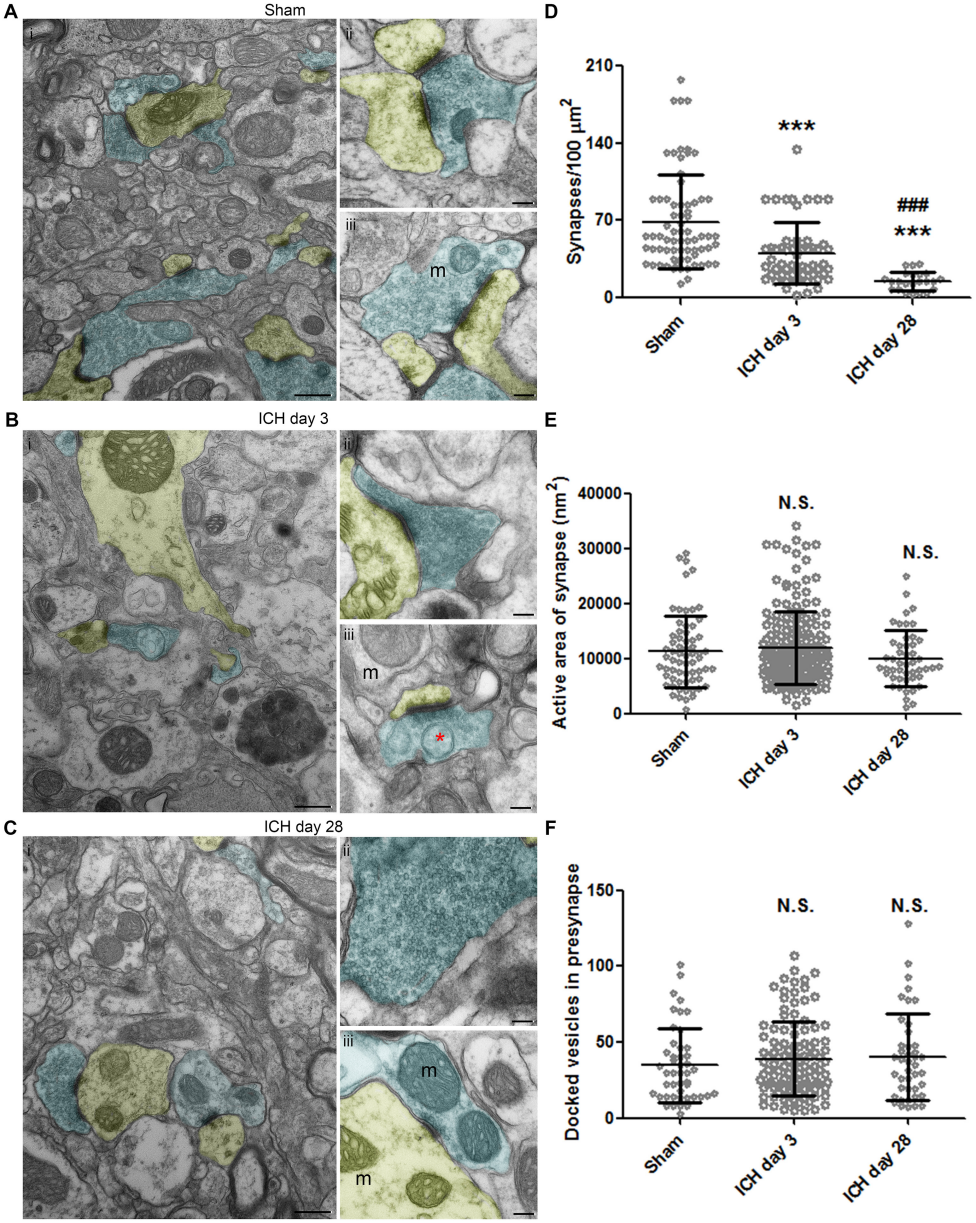
Synaptic changes at 3 and 28 days after intracerebral hemorrhage (ICH). Synapses in the ipsilateral striatum of mice after sham procedure **(A)**, at 3 days after ICH **(B)**, and at 28 days after ICH **(C)**. Presynapses with transmitter vesicles are shown in blue; post-synapses are shown in yellow. The red asterisk in **(B)** indicates an autophagosome. **(D)** Quantification of synapse density at various time points after ICH. The number of synapses was normalized by 100-μm^2^ fields. Number of fields: sham, *n* = 71 fields; ICH day 3, *n* = 55 fields; ICH day 28, *n* = 23 fields. **(E)** Quantification of active area in synapses at various time points after ICH. Number of synapses: sham, *n* = 65; ICH day 3, *n* = 180; ICH day 28, *n* = 48. **(F)** Quantification of docked vesicles in synapses at various time points after ICH. Number of synapses: sham, *n* = 46; ICH day 3, *n* = 113; ICH day 28, *n* = 42. Results are shown as scatter graphs (mean ± SD). ^***^p < 0.001 vs. sham; ^###^*p* < 0.001 vs. ICH day 3; N.S., not significance. One-way ANOVA followed by Dunn's *post-hoc* analysis. Scale bars: **(Ai,Bi,Ci)** 500 nm; **(Aii–iii,Bii–iii,Cii–iii)** 100 nm. m: mitochondria. *n* = 6 animals per group. In **(D)**, each dot represents one field; in **(E,F)**, each dot represents one synapse.

In the sham animals, the number and density of synapses was normal under TEM, and transmitter vesicles were visible in the presynaptic axons (Figure 5A). At 3 days post-ICH, fewer synapses were present, and the presynaptic axons contained fewer transmitter vesicles (Figure 5B). Organelles and even autophagosomes were seen in the presynaptic axons and post-synaptic dendrites (Figures 5Bii,iii). At day 28 post-ICH, abnormal synapses were still present, and the presynaptic axons contained either substantially more or substantially fewer vesicles than normal (Figure 5C). Surprisingly, swollen mitochondria could still be seen in axons and dendrites at 28 days, indicating the continued presence of unhealthy neurons (Figure 5Ciii). To further investigate synaptic degradation quantitatively, we calculated the synapse density (Figure 5D). Compared to that in sham animals, the number of synapses per 100 μm^2^ decreased at day 3 and further declined at day 28 (Figure 5D). Besides synapse density, area of active zone and number of docked presynaptic vesicles are the common measurements of synaptic degradation. As shown in Figures 5E,F, there was a trend toward difference between sham and ICH animals, but we found no statistical significance among groups (Figures 5E,F).

### Axonal demyelination at 28 days after ICH

Using Luxol fast blue staining, we have shown that white matter fiber tracts in the ipsilateral corpus callosum and internal capsule are markedly damaged at day 3 (42). The continued decrease in fractional anisotropy values measured by magnetic resonance imaging (MRI) at these two locations indicates that white matter injury persisted until day 28 after ICH (42). Similarly, the myelin basic protein expression was markedly decreased on day 7 and had not returned to basal level at day 28 (24). Importantly, the Olig2^+^ cells (oligodendrocytes) that were recruited and repopulated in the perihematoma region at early time points might contribute to remyelination at late disease stages (24).

**Figure 6:**
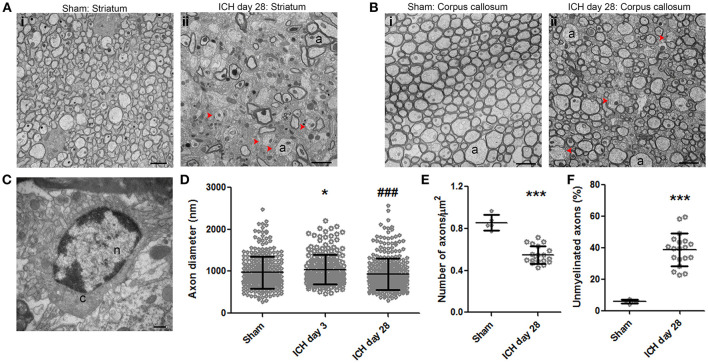
Demyelination of axons at 28 days after intracerebral hemorrhage (ICH). **(A)** Striatum of sham mouse (i). Note the relative uniformity in size and shape of the axons, as well as the healthy mitochondria and normal myelination. At 28 days after ICH (ii), the axons (a) of the striatum have lost their uniformity in size and shape. Additionally, many axons lack a myelin sheath; several of these demyelinated axons are indicated with red arrowheads. **(B)** The healthy corpus callosum of a sham mouse (i). Note the uniform size and shape of the axons (a). At 28 days after ICH (ii), the axons exhibit a significant loss of uniformity in size and shape. As in the striatum, several axons lack myelin (red arrowheads). **(C)** Striatum of a mouse at 28 days post-ICH shows an oligodendrocyte. **(D)** Quantification of axon diameter in the ipsilateral striatum at various time points after ICH and in sham animals. Number of axons: sham, *n* = 327; ICH day 3, *n* = 208; ICH day 28, *n* = 361. Results are shown as scatter graphs (mean ± SD); each dot represents one axon. ^*^p < 0.05 vs. sham; ^###^*p* < 0.001 vs. ICH day 3. One-way ANOVA followed by Dunn's *post-hoc* analysis. **(E)** Quantification of axonal density in the ipsilateral striatum of sham and ICH mice at 28 days. The number of axons was normalized by 1-μm^2^ fields. Number of fields: sham, *n* = 5 fields; ICH day 28, *n* = 19 fields. Results are shown as scatter graphs (mean ± SD); each dot represents one field. ^***^p < 0.001 vs. sham by two-tailed Student's *t*-test followed by Welch's correction. **(F)** Quantification of unmyelinated axons in the ipsilateral striatum of sham and ICH mice at 28 days. Axons were counted in 230-μm^2^ fields: sham, *n* = 5 fields; ICH day 28, *n* = 19 fields. Results are shown as scatter graphs (mean ± SD); each dot represents one field. ^***^p < 0.001 vs. sham by two-tailed Student's *t*-test followed by Welch's correction. Scale bars: **(A,B)** 2 μm; **(C)** 500 nm. n, nucleus; c, cytoplasm; a, axon. *n* = 6 animals per group.

Using TEM, we found that, in the ipsilateral striatum of injured animals, axons showed a clear pattern of demyelination and a loss of uniformity in size and shape (Figure 6Aii) when compared with axons of the sham animals (Figure 6Ai). Interestingly, similar demyelination was seen in the ipsilateral corpus callosum of injured animals (Figure 6Bii), whereas sham animals exhibited healthy, uniform myelinated axon patches (Figure 6Bi). Between axons, we observed oligodendrocytes, which are responsible for producing myelin and remyelinating axons. Figure 6C presents an oligodendrocyte with a classic morphology. To further investigate the pathologic changes in axons after ICH, we first quantified the axon diameter at different time points. We found that the axon diameter was significantly greater in animals at day 3 after ICH than in sham animals (Figure 6D), indicting axonal swelling and degeneration in the acute phase of ICH, consistent with our findings in Figure 3. However, we failed to detect statistical differences between the sham group and the 28-day ICH group (Figure 6D). We further investigated the density of axons and the percentage of demyelinated axons. In the ipsilateral striatum, the number of axons in a 1 μm^2^ area on day 28 after ICH was significantly decreased compared to that in sham animals (Figure 6E). In addition, the ratio of unmyelinated to myelinated axons in the ipsilateral striatum indicated a higher percentage of unmyelinated axons in ICH animals than in sham animals at 28 days (Figure 6F).

## Discussion

Mouse models, particularly those in which hemorrhage is induced with collagenase, are important tools for studying ICH and have been used by many researchers (43). In this study, we examined the ultrastructural features of mouse brain after collagenase-induced ICH. The collagenase- and blood-injection animal models of ICH share pathologic similarities in terms of tissue damage from blood components; however, the collagenase injection model does have features of hematoma expansion and increased intracranial pressure that resemble clinical ICH (1, 42, 44). Using TEM, we demonstrated axonal demyelination and degeneration, as well as the presence of dystrophic neurites in the axons. Various types of cell death, including necrosis, ferroptosis, and autophagy, were observed in neuronal somas at the same perihematoma region (1 mm from hematoma margin). Additionally, microglia and macrophages exhibited activation and phagocytosis at 3 days.

Our findings partially support previous research in both animal (22, 23) and human brain samples (12, 16). In a rat collagenase injection model, apoptosis was observed at 24 h post-ICH, as evidenced by large numbers of TUNEL-positive cells that had morphologies indicative of apoptosis (45). In a rat blood injection model, TUNEL-positive cells appeared as early as 6 h after ICH (46). Cell death via apoptosis has been replicated by a number of different studies involving human samples, with apoptotic cells present in specimens obtained 1, 2, and 5 days after onset of symptoms (12, 47). Increasing complement activation and subsequent apoptotic cell clearance has been suggested as a therapeutic target for ICH in humans (47). However, at 3 and 6 days post-ICH, we did not observe classic apoptotic cells, which exhibit chromatin condensation and apoptotic bodies. It is possible that the classic morphology of apoptosis is present only at time points other than 3 and 6 days, that apoptotic cells might appear in the middle of the hematoma or in brain regions at least 1 mm from the margin of hematoma), or that TUNEL staining only indicates DNA damage and is not specific for apoptosis, as some have suggested (48, 49).

We assessed neuronal autophagy after ICH, as we and others have reported (5, 18, 50, 51). Using Western blotting to measure the ratio of LC3-II to LC3-I, He et al. showed that autophagy appeared at 3 days and peaked after 7 days in a rat model (18). Hu et al. reported that neuronal autophagy appeared at 3 days in a thrombin injection model (51). Necrosis has been identified as another major form of cell death in various mouse and human studies (12, 15). Necrosis was observed by electron microscopy at 24 h after ICH in mice (15). We found that, unlike apoptosis, different degrees of necrosis were present in brain regions within 1 mm of the hematoma margin at 3 and 6 days and that most of the dying cells were necrotic. Interestingly, contrary to our TEM data, perfusion computed tomography (52) and other imaging-based (53, 54) human studies failed to detect significant necrosis and ischemia in the perihematoma region in the hyperacute and acute stages. This discrepancy may be due to differences between species or different sensitivity analysis methods for detecting neuronal death.

Ferroptosis (35) is a recognized, iron-dependent form of cell death (5, 20). Although we did not inject ferrous iron into the mouse brain to identify ferroptosis specifically, we and others have shown that both FeCl_2_ and hemoglobin can induce ferroptosis in organotypic slice cultures (5) and in cultured primary neurons (20). Similar to our previous report, ferroptosis was observed at both 3 and 6 days post-ICH. At 28 days post-ICH, we did not find any increase in the frequency of smaller mitochondrial area, but the increased frequency of larger mitochondrial area persisted. Given that the presence of shrunken mitochondria is the only gold standard by which to identify ferroptosis with TEM, and that it is impossible to define shrunken mitochondria without quantifying a large number of cells *in vivo*, we were unable to identify which cells were undergoing ferroptosis. Thus, we can only assume that the ferroptotic neurons were mixed with necrotic and autophagic neurons in brain regions within 1 mm of the margin of the hematoma. More research is needed to clarify the molecular mechanisms of ferroptosis after ICH.

The innate immune response is increased after ICH (9). Macrophage and microglial staining increased after ICH in mice (22, 39). Dahnovici et al. reported that the number of phagocytic macrophages increased 7–8-fold in areas surrounding hematoma in humans (55). Microglia are the first cells to respond after ICH (37, 56). Monocyte-derived macrophages also contribute to neuroinflammation, debris-clearance, hematoma-clearance, and brain repair, similar to resident microglia (1). We are the first to show erythrophagocytosis by microglia and macrophages after ICH using TEM. We observed cell debris, including autophagosomes, engulfed by phagocytes. Although microglial autophagy occurs after ICH and contributes to microglial activation (57), we tend to think that it would be microglial phagocytosis of autophagic neurons rather than microglia themselves undergoing autophagy. According to reports in the literature, microglial activation peaks from 1 to 3 days after ICH and is negligible after 7 days (58). Additionally, monocyte-macrophage infiltration peaks at day 1 and is mostly gone after 7 days (59). Consistent with these data, which were obtained by immunostaining and flow cytometry, we observed phagocytosis by microglia and macrophages mainly at day 3. It was less frequent at day 6 and mostly absent at day 28. We observed white blood cells in blood vessels at day 3, but not at day 6.

Synaptic changes have been noted in human ICH brain (60). In animals, electron microscopy showed loss of synapses 1 day after ICH (25). We showed a significant loss of synapses on day 3, consistent with findings by Nguyen et al., who used Golgi-Cox stain to assess dendritic injury in the subacute phase post-ICH (41). Additionally, we observed a further decrease in synaptic density on day 28. However, we failed to detect any significant changes in mean active area or number of vesicles in a single synapse. Quantification in serial sections may be needed to detect these changes. Research should explore how to enhance synaptogenesis after ICH, as one study indicated that motor skill training may be beneficial (61).

Axonal degeneration after ICH has been observed in human (16) and mouse (23, 62) brain samples. Moreover, ICH patients with a low Glasgow Coma Scale score exhibit clear loosening of myelin sheaths around axons (16). We showed significant axonal degeneration in the striatum, which is the predominant location of hematoma in humans. Importantly, using TEM, we detected demyelination in the ipsilateral corpus callosum, which supports our previous findings with MRI and other histologic methods that ICH produces white matter injury (17, 42). At 28 days post-ICH, we found a significant decrease in the density of all axons and the ratio of unmyelinated to all axons (myelinated plus unmyelinated) in striatum, but we failed to see demyelination at 3 or 6 days statistically (data not shown). We did see an increase in myelin sheath thickness at 3 and 6 days post-ICH (data not shown), a finding that is consistent with our data showing that axonal diameter was increased on day 3 compared to that in the sham group. In the brain, oligodendrocytes are responsible for myelination of axons (63). Notably, we observed a proliferation of oligodendrocytes after ICH (data not shown), supporting the findings of a recent study in rats (24).

This study has limitations. Ultrastructural changes at earlier time points after ICH need to be investigated, as Garcia et al. found that abnormalities of neuronal soma can be observed even within hours after ischemic injury (64). Second, parallel histologic experiments need to be performed to support the findings from TEM. Lastly, the blood-injection ICH model should be used and the results compared to those from the collagenase model.

## Conclusion

After collagenase-induced ICH, mouse brain showed significant ultrastructural changes marked by cell death, axonal degeneration, and a robust inflammatory response. Increased activation of the innate immune response could be a subject of future research, as the role of inflammation is under debate.

## Author contributions

QL and JW designed the experiments. QL and XC collected the data. QL, AW, and FD analyzed the data. QL, AW, and JW wrote the manuscript. QL, XLa, XH, XLi, JW, XC, WZ, DH, and JW revised the manuscript.

### Conflict of interest statement

The authors declare that the research was conducted in the absence of any commercial or financial relationships that could be construed as a potential conflict of interest.
