# Corrigendum: Ultrastructural characteristics of neuronal death and white matter injury in mouse brain tissues after intracerebral hemorrhage: coexistence of ferroptosis, autophagy, and necrosis

**DOI:** 10.3389/fneur.2024.1385719

**Published:** 2024-03-01

**Authors:** Qian Li, Abigail Weiland, Xuemei Chen, Xi Lan, Xiaoning Han, Frederick Durham, Xi Liu, Jieru Wan, Wendy C. Ziai, Daniel F. Hanley, Jian Wang

**Affiliations:** ^1^Department of Anesthesiology and Critical Care Medicine, Johns Hopkins University School of Medicine, Baltimore, MD, United States; ^2^Department of Biochemistry and Molecular Biology, School of Basic Medical Sciences, Capital Medical University, Beijing, China; ^3^Advanced Innovation Center for Human Brain Protection, Beijing, China; ^4^Department of Human Anatomy, College of Basic Medical Sciences, Zhengzhou University, Zhengzhou, China; ^5^Department of Neurology, Johns Hopkins University School of Medicine, Baltimore, MD, United States

**Keywords:** cell death, intracerebral hemorrhage, synapse, transmission electron microscopy, white matter injury

In the published article, there was an error in Figure 2Ci as published. The wrong image was inadvertently used. The corrected Figure 2 appears below.

**Figure 2:**
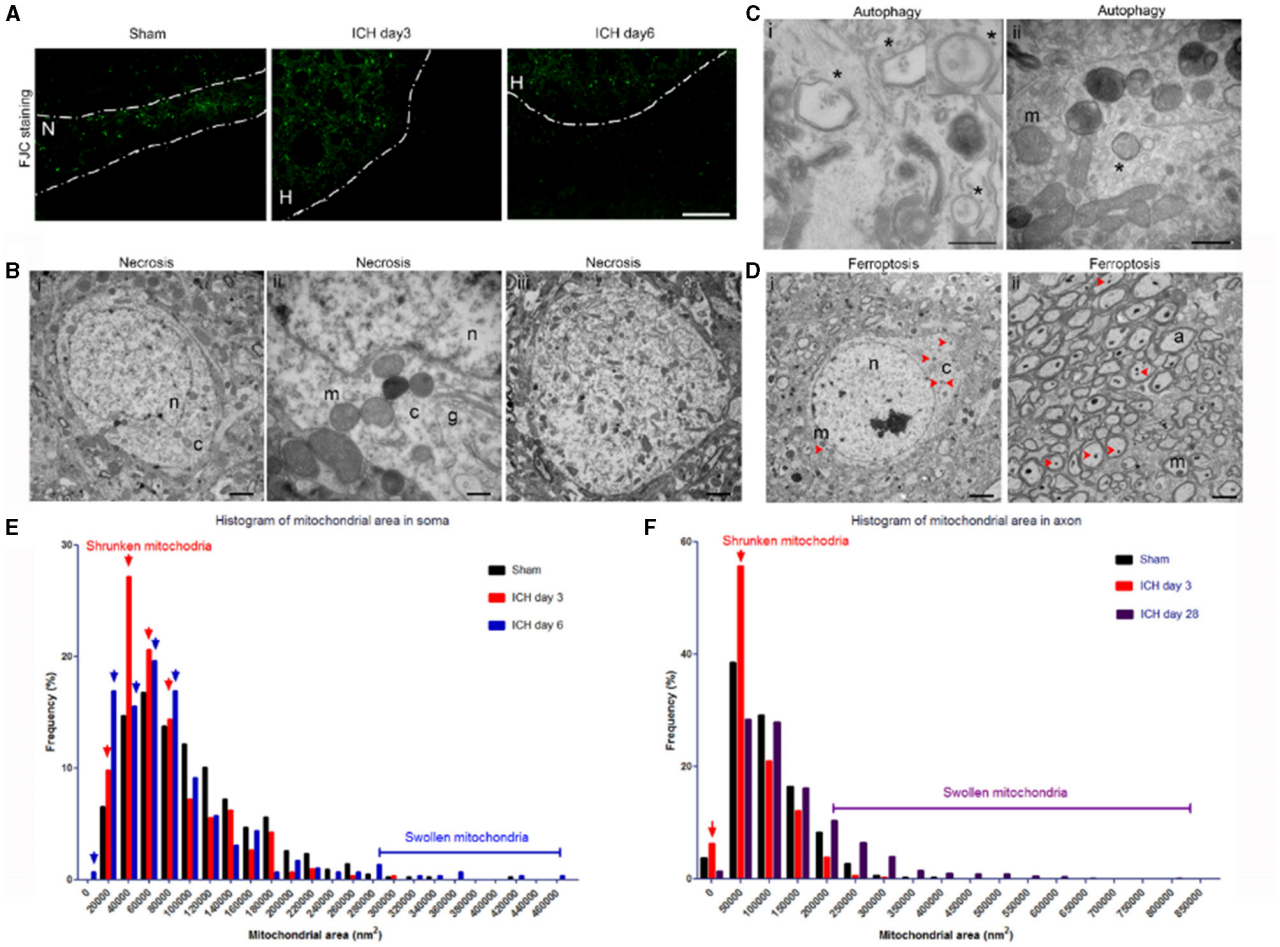
Mixed forms of neuronal death after intracerebral hemorrhage (ICH). **(A)** Fluoro-Jade C histology shows degenerating neurons in the perihematoma region at 3 and 6 days post-ICH or along the needle track in the striatum of the sham-operated mice. Dashed lines indicate the margin of hematoma. N, needle track; H, hematoma. **(B)** Necrotic neuronal soma (i–iii) show loss of a distinct nuclear membrane, enlarged mitochondria, and changes in chromatin structure at 3 days post-ICH. **(C)** Autophagy in neuronal soma at 3 days post-ICH; autophagosomes are labeled with asterisks. Inset shows a high-power image of an autophagosome. **(D)** Neuronal soma and axons show signs of ferroptosis with evidence of shrunken mitochondria (red arrowheads) at 3 days post-ICH. **(E)** Quantification of mitochondrial area frequency in neuronal somas at various time points after ICH. Arrows indicate increased frequency of shrunken mitochondria on days 3 and 6. Number of mitochondria: sham, *n* = 429; ICH day 3, *n* = 306; ICH day 6, *n* = 296. **(F)** Quantification of mitochondrial area frequency in axons at various time points after ICH. Arrows indicate increased frequency of shrunken mitochondria on day 3. Number of mitochondria: sham, *n* = 433; ICH day 3, *n* = 314; ICH day 28, *n* = 603. Scale bars: **(A)** 100 μm; **(Bi,iii, D)** 2 μm; **(Bii, Cii)** 500 nm. n, nucleus; c, cytoplasm; m, mitochondria; a, axon. *n* = 6 animals per group.

In the published article, there was an error in the Funding statement. The NSFC (U1704166), the Henan Province Science and Technology Cooperation Project (No. 182106000061) or the NIH grants (R01NS078026, R01AT007317, R56NS096549, R21NS101614, R21NS102899 and UG3NS106937) did not support this work. The correct Funding statement appears below.

